# Medical and Philosophical News

**Published:** 1781-07

**Authors:** 


### SECTION III.

### Medical and Philosophical News.

THE Oeconomical Society at Berne in Switzerland have propoſed the following ſubject for a prize of ſixty ducats, "What are the "vapours produced by the fermentation of thoſe "vegetables which are inflammable, and by "what circumſtances are they produced and "rendered inflammable?" The diſſertations are to be written in Latin, German, French, Engliſh, or Italian, and ſent to M. de Haller de Roche, ſecretary to the ſociety, on or before the the 31ſt of December 1782.

---

The library, chemical and philoſophical inſtruments, foſſils, &c. belonging to the late Dr. Lewis of Kingſton, have lately been ſold in London.

don. The library contained a great number of fcarce books in chemiftry and pharmacy, but the moft valuable part of it was a collection of manufcripts relating chiefly to philofophical fubjects, phyfic, chemiftry, natural hiftory, arts, and manufactures, and moft of them written by a Mr. Chifholm, who for upwards of thirty years was employed by Dr. Lewis for that purpofe, and who, befides being a good chemift, is well acquainted with the French, Swedifh, and German languages. We fhall here mention fome of the moft valuable lots in this collection with the prices they fold at.—1. Bergman's phyfical hiftory of the earth. From the Swedifh. 2 vol. 8vo. £.3 13 6.—II. Bergman's method of examining the contents of waters, extracted from his feveral papers in the Swedifh acts, 7 s. —III. Select papers, and abridgments of papers, medical, phyfical, metallurgical, chemical, and in various branches of natural hiftory, arts and manufactures, tranflated from the memoirs of the Royal Academies of Paris and Berlin, 2 vols. folio, 4 vols. 4to. 3 vols. 8vo. £.4 4.—IV. The art of brewing; five fections of which only are finifhed; and thefe are as follows, 1. Hiftory of fermentation: 2. Of malt: 3. Of water; purifying, hardening, &c.

4. Of meafuring heat: 5. Of determining the ftrength of worts, £.1 11 6.—V. Hoffman's medical works abridged, with additions under each difeafe, from later writers, 2 vols. 8vo. £.3 10 6.—VI. Chemical lectures read to his prefent Majefty, when Prince of Wales, at Kew, 4to. Ditto to their Royal Highneffes the Duke and Duchefs of Gloucefter at Kingfton, 4to. £.1 17.—VII. Collections on iron from all the experimental writers that could be procured; from the information of the moft experienced workmen, and from obfervations at the works; with a view to a general hiftory of iron and its manufactures. 1 vol. fol. 2 books 4to. 4 books 8vo. Confiderable parts of the faid hiftory digefted; but wanting fome further experiments and informations for completing them, 6 books 4to. £.21 10 6.—VIII. Collections on the hiftory of tin, and the arts depending thereon, from the beft experimental writers, German, Swedifh, and French, with the procefs of making tin plates, by a manufacturer, 8vo. £.1 18.—IX. Synoptic tabular views of the properties of the different metallic bodies, fol. £.2 4.—X. Hellot's art of dying, from the French, with additions, 2 vols. 8vo. Extracts from Stahl's Ars tinctoria fundamentalis, 8vo. £.18 7 6.—XI. Tranflations of entire entire papers, abridgments and abſtracts of all the papers on chemical, medical, and phyſical ſubjects contained in the Swediſh acts, or tranſactions of the Royal Academy at Stockholm, from 1739 to 1776 incluſive. Tranſlated from the Swediſh, with a table. 7 vols. 8vo. £.31 10 6. —XII. Pott's *Lithogeognoſia*, 4to. Chemical eſſays, 4 vols. 8vo. and other Chemical works by the ſame author. Tranſlated from the German. £.2 5.——The whole collection of manuſcripts, conſiſting of 133 lots, ſold for about 200 guineas.

---

We have lately received from Stockholm a paper, in the Swediſh language, written by the ingenious Mr. Scheele, and deſcribing his method of preparing *mercurius dulcis*, *via humida*, and likewiſe a new green colour. His account of the firſt of theſe proceſſes is as follows:

"Half a pound of quickſilver, and the ſame quantity of pure aqua fortis, are to be put into a ſmall veſſel with a long neck, the mouth of which is to be covered with paper. The veſſel is then to be placed in a warm ſand bath, and after a few hours, when the acid affords no ſigns of its acting any longer on the quickſilver, the fire is to be increaſed to ſuch a degree that the ſolution may nearly boil. This heat is to be continued for three or four hours, taking care to move the veſſel from time to time, and at laſt the ſolution is to be ſuffered to boil gently for about a quarter of an hour. In the mean while we are to diſſolve four ounces and a half of fine common ſalt in ſix or eight pints of water. This ſolution is to be poured boiling into a glaſs veſſel, in which the abovementioned ſolution of quickſilver is to be mixed with it, gradually, and in a boiling ſtate alſo, taking care to keep the mixture in conſtant motion. When the precipitate is ſettled, the clear liquor is to be drained from it, after which it is to be repeatedly waſhed with hot water till it ceaſes to impart any taſte to the water. The precipitate obtained by this method is to be filtred, and afterwards dried by a gentle heat.

" It might be ſuppoſed, that when the nitrous acid ceaſes to effervefce with the mercury, it is ſaturated with it; but this is far from being the caſe; the acid, when the heat is increaſed, being ſtill able to diſſolve a conſiderable quantity of it, with this difference, however, that the quickſilver at the beginning of the proceſs is calcined by the acid, but afterwards is diſſolved by it in a metallic form. In proof of this we may obſerve, ferve, that not only more elaftic vapour arifes, but alfo that by adding either fixed or volatile cauftic alkali we obtain a black precipitate, whereas when the folution contains only calcined quickfilver, the precipitate becomes yellow by fuch an addition. If this black precipitate is gently diftilled, it rifes in the form of quickfilver, leaving a yellow powder, which is in fact that part of the mercury that in the beginning of the operation was calcined by the nitrous acid.

" The boiling of the folution for about a quarter of an hour is neceffary, in order to keep the *mercurius nitratus* in a diffolved ftate, it being much difpofed to chryftallize. In general, fome of the mercury remains undiffolved, but it is always better to take too much than too little of it, becaufe the more metallic fubftance the folution contains, the more mercurius dulcis will be obtained.

" It is neceffary to pour the mercurial folution into the folution of falt by a little at a time, and cautioufly, fo that no part of the undiffolved quickfilver may pafs along with it. Two ounces of common falt are fufficient to precipitate all the mercury, but then it may eafily happen that fome fuperfluous mercurius corrofivus attaches itfelf to this precipitate, which the water alone is incapable of of feparating completely. This is undoubtedly the reafon why mercurius precipitatus albus is always corrofive. I have found that common falt poffeffes the fame quality as fal ammoniac, viz. that of diffolving a great quantity of mercurius corrofivus. I therefore employ four ounces and a half of common falt in order to get the mercurius corrofivus entirely feparated.

" If we confider the manner in which mercurius dulcis is obtained in the dry way, by fublimation, we fhall not find it difficult to give the rationale of this new procefs.

" Mercurius corrofivus albus is a middle falt, confifting, as is well known, of marine acid combined with calx of mercury. This falt is capable of diffolving a good deal of quickfilver in a metallic form, but for this purpofe the moft minute particles of each muft be reciprocally mixed. This happens, when by means of heat, they are both converted into vapour. The fame thing occurs in the abovementioned procefs. The folution firft fpoken of contains the calx mercurii and quickfilver divided into the moft minute particles. If to this folution we add marine acid, or (to fave expence) common falt, the marine acid will unite with the calx of mercury, and the refult of this union will be a true mercurius curius corrosivus albus ; and as the solution contains quicksilver in its metallic state, this will immediately attract as much of the mercurius corrosivus as is necessary to saturate it, and by this means a real mercurius dulcis will be produced, which, from its being insoluble, will be immediately precipitated.

"The following facts are proofs that this precipitate is a good mercurius dulcis, 1st, It is entirely tastleless. 2dly, I have sublimed it and examined what ascended in the beginning, and which ought to have been corrosive, if the precipitate had contained any thing of that nature; it being well known that mercurius corrosivus ascends sooner than mercurius dulcis, whereas through the whole of the sublimation what arose was a pure mercurius dulcis, exactly like that which is obtained in the common manner. 3dly, I have mixed this precipitate with one fourth part of quicksilver and sublimed it, upon a supposition that if it contained too much mercurius corrosivus it would be able to unite with more quicksilver; but so far was this from being the case, that the quicksilver was not diminished in weight by the experiment. 4thly, It is known that caustic alkalis and lime water give mercurius dulcis a black colour. The same thing happened with mine. The black colour is no other than quickfilver divided into very fine particles.

"That the procefs I have been defcribing is more advantageous than that which is ufually adopted, I cannot doubt ; becaufe, in the firft place, this mercurius dulcis can be prepared with lefs difficulty, with lefs expence, and without employing corrofive fublimate. 2dly, As there can be no danger of its being in any degree corrofive, provided it be fufficiently edulcorated, it may always be given with fafety. 3dly, The operator is not expofed to that noxious duft which in the old method arifes during the trituration of the corrofive fublimate and quickfilver. 4thly, This is much finer than the common mercurius dulcis, it being impoffible to make the latter equal to it in this refpect, however long it may be triturated."

To this account we may add that Dr. Elliot, having undertaken, at the requeft of the fociety, to repeat Mr. Scheele's procefs, finds that a pound of mercury, and the fame quantity of aqua fortis, treated in the manner above defcribed, yield thirteen ounces and a half of calomel. In our next number we fhall infert Mr. Scheele's method of preparing a new green colour.

---

From the report of the SMALL POX SOCIETY at CHESTER, dated March 27,1781. It appears that at the beginning of May 1780,when the ſmall pox prevailed in that place, the town was divided into ſix diſtricts, each under the care of an inſpector; during that month the diſtemper was in twenty-five families, and in five out of the ſix diſtricts. In all theſe places, except one, the contagion was totally extinguiſhed on the 17th of June. In this diſtrict the diſtemper was only checked, through the imperfect execution of the regulations, but not ſtopt, till the 9th of December, and till theſe irregularities were corrected. During this period the ſmall pox appeared in two diſtant parts of the town, but the contagion was ſtopt in both places without infecting a ſecond patient. Since that time it has been brought into Cheſter, twice from Liverpool, and once from Coventry. The rules of prevention, in two inſtances, have preſerved from the contagion two perſons, never infected, in the ſame houſe: and in another inſtance three perſons, never infected, in the ſame family, with a patient in the natural ſmall pox, during the whole diſeaſe. At the time of dating the report there was not a ſingle patient with the natural ſmall pox in Cheſter.—The annual expence of this in-

ſtitution amounts to about 100 guineas. The inſpectors are authorized to give a reward to the perſon who firſt informs them that a freſh family is infected.

---

The following new works are in the preſs; viz. 1. Obſervations on Diſorders of the Nerves. By Alexander Thomſon, M. D. 2. Elements of the Branches of Natural Philoſophy connected with Phyſic. By John Elliot, M. D.

